# Occupant Interactions and Effectiveness of Natural Ventilation Strategies in Contemporary New Housing in Scotland, UK

**DOI:** 10.3390/ijerph120708480

**Published:** 2015-07-21

**Authors:** Tim Sharpe, Paul Farren, Stirling Howieson, Paul Tuohy, Jonathan McQuillan

**Affiliations:** 1 Mackintosh Environmental Architecture Research Unit, Glasgow School of Art, 167 Renfrew Street, Glasgow G3 6RQ, UK; 2 ASSIST Design Architects, 100 Kerr Street, Glasgow G40 2QP, UK; E-Mail: paulf@assist.co.uk; 3 ESRU/Department of Architecture, University of Strathclyde, James Weir Building, 75 Montrose Street, Glasgow G1 1XJ, UK; E-Mails: s.howieson@strath.ac.uk (S.H.); paul.tuohy@strath.ac.uk (P.T.); 4 Anderson Bell Christie, 382 Great Western Road, Glasgow G4 9HT, UK; E-Mail: jonathanmcquillan@andersonbellchristie.com

**Keywords:** ventilation, IAQ, housing, bedrooms, CO_2_, building regulations, trickle ventilators

## Abstract

The need to reduce carbon emissions and fuel poverty has led to increased building envelope air tightness, intended to reduce uncontrolled ventilation heat losses. Ventilation strategies in dwellings still allow the use of trickle ventilators in window frames for background ventilation. The extent to which this results in “healthy” Indoor Air Quality (IAQ) in recently constructed dwellings was a concern of regulators in Scotland. This paper describes research to explore this. First a review of literature was conducted, then data on occupant interactions with ventilation provisions (windows, doors, trickle vents) gathered through an interview-based survey of 200 recently constructed dwellings, and measurements made on a sample of 40 of these. The main measured parameter discussed here is CO_2_ concentration. It was concluded after the literature review that 1000 ppm absolute was a reasonable threshold to use for “adequate” ventilation. The occupant survey found that there was very little occupant interaction with the trickle ventilators e.g., in bedrooms 63% were always closed, 28% always open, and in only 9% of cases occupants intervened to make occasional adjustments. In the measured dwellings average bedroom CO_2_ levels of 1520 ppm during occupied (night time) hours were observed. Where windows were open the average bedroom CO_2_ levels were 972 ppm. With windows closed, the combination of “trickle ventilators open plus doors open” gave an average of 1021 ppm. “Trickle ventilators open” gave an average of 1571 ppm. All other combinations gave averages of 1550 to 2000 ppm. Ventilation rates and air change rates were estimated from measured CO_2_ levels, for all dwellings calculated ventilation rate was less than 8 L/s/p, in 42% of cases calculated air change rate was less than 0.5 ach. It was concluded that trickle ventilation as installed and used is ineffective in meeting desired ventilation rates, evidenced by high CO_2_ levels reported across the sampled dwellings. Potential implications of the results are discussed.

## 1. Introduction

The need to reduce energy consumption and carbon emissions from housing [1] has driven revisions of the Building Regulations in the UK [2,3]. As transmission heat losses were decreased, the relative importance of intended and unintended ventilation losses increased. To address unintended ventilation losses more demanding requirements for building envelope air tightness have been introduced, the impact of which has been characterised by various researchers, notably BRE [4,5,6] and Leeds Metropolitan University [7,8]. These researchers reported increasing air tightness for more recent dwellings as measured by blower door tests: for example pre-2002 stock had a mean value of 12 m^2^/m^3^·h @ 50 Pa and range from 2 to 22 m^2^/m^3^·h @ 50 Pa; post-2002 had a mean and range of 10 m^2^/m^3^·h @ 50 Pa and 4 to 14 m^2^/m^3^·h @ 50 Pa; and post 2006 has a mean and range of 6 and 1 to 12 m^2^/m^3^·h @ 50 Pa. A mean measured value of 4 m^2^/m^3^·h @ 50 Pa was associated with the post-2009 dwellings monitored in this work (Section 4).

There are emerging concerns that ventilation and IAQ is declining to levels that could negatively impact on the health of the occupants [9,10]. Regulations in Scotland continue to allow natural ventilation provision through trickle ventilators set into window frames to provide adequate background air changes with only intermittent mechanical ventilation in wet rooms for moisture and odour control. The Building Standards (Scotland) Regulations [11] set an upper limit for air tightness of 10 m^2^/m^3^.h @ 50 Pa with higher than prescribed standards being routinely achieved [12].

Although research commissioned by the Building Standards Directorate (BSD) in 2012 indicated that trickle ventilators could provide acceptable levels of ventilation [13] the work assumed that trickle ventilators and internal doors were all kept open, and did not consider practical factors such as the obstruction of trickle ventilators by blinds and curtains *etc.* Subsequent research conducted on contemporary housing in Scotland suggested that these findings do not reflect real world conditions [14] in which occupants’ behaviour towards window and trickle ventilation is uncertain, and privacy, security, external noise, fire regulations in flats, may mean that trickle vents and internal doors remain closed.

Recognising that the reality of the current situation in recently constructed dwellings was uncertain, the Scottish Building Standards organisation commissioned research to investigate. This research was to include a review of literature, gathering of data on how occupants are interacting with ventilation provisions (windows, doors, trickle vents) across 200 dwellings, and measurements of IAQ in a sample of 40 of these. Some highlights from this research are documented in this paper.

### 1.1. Previous studies: CO_2_, Ventilation and Health

Many previous studies use CO_2_ levels as a proxy for ventilation rates and an indicator of IAQ. Humans are the primary source of CO_2_ in the indoor environment [15,16] and are also a source of heat, moisture, smells and other bio-emissions which can influence perceived IAQ. Levels of CO_2_ have long been used as a basis for ventilation design and control [17] as high levels are a good surrogate for human emitted bio-effluents (*i.e.*, odours) that are considered undesirable. The concentration levels of CO_2_ depend on the number of occupants in a given area, their activity level, and the rate at which the local air is diluted and replaced by fresh air (ventilation rate).

There is a general acceptance that CO_2_ can keep “bad company” and that absolute levels above 1000 ppm (>500–600 above ambient) are indicative of poor ventilation rates as originally identified by Pettenkoffer [18]. The provenance of this is well evidenced [19] and corresponds in standard situations with the generally recommended ventilation rate of 8 L/s per person [20]. Associations between health and CO_2_ levels have been found in office buildings [21,22] and a study by Batterman and Peng [23] identified associations between CO_2_ levels and total volatile organic compounds (TVOCs).

Both the Chartered Institute of Building Services Engineers (CIBSE) and American Society Of Heating, Refrigerating, and Air-Conditioning Engineers (ASHRAE) recommendations for ventilation rates are based on or around 1000 ppm and 8 L/s/person (circa 30 m^3^/h/person) [24,25,26]. CO_2_ threshold levels of 1000 ppm in communal areas such as offices and schools have been set as indicators of sufficient per person ventilation rates [27].

Although less research has been undertaken in housing, a meta-analysis carried out by Wargoki identified associations between CO_2_ levels and health and concluded “*The ventilation rates above 0.4 h^−1^* *or CO_2_ below 900 ppm in homes seem to be the minimum level to protect against health risks based on the studies reported in the scientific literature*.” [28]. The latest guidance from ASHRAE member organisations supports the translation of the 1000 ppm limits to housing both for living areas and for master bedrooms [29].

CO_2_ does not have the same relationship with pollutants from non-human sources that it does with human emissions. Off-gassing from building materials, furnishings, cooking, cleaning and electrical products may be independent of occupancy [30] e.g., Offermann found low CO_2_ levels in conjunction with dangerously high levels of formaldehydes [31]. However where there are low ventilation rates there are relatively higher pollutant concentrations from both human and non-human sources, and so using CO_2_ as a proxy for ventilation rate in situations where occupancy is known is a justifiable approach. A recent study by Ramalho et al [32] found that CO_2_ was positively and significantly associated with a range of pollutants, but concluded that even with good ventilation conditions the control of pollutant sources remains necessary. A study in Korea [33] reported that increases of indoor CO_2_ concentrations as low as 564 ppm above external levels were associated with wheezing attacks in children with a history of asthma.

The conclusion from this review was that CO_2_ could be used as an indicator of ventilation rate in situations where occupancy is known, and that the generally accepted desirable maximum absolute CO_2_ level in occupied spaces is 1000 ppm.

### 1.2. IAQ and Ventilation in Building Regulations, and Associated Studies

The building regulations in the UK are geared to prescriptive measures to ensure “adequate ventilation” but there are currently no mandatory standards covering IAQ with respect to specific CO_2_ levels or toxic pollutants. Harrison [34] has reviewed the guideline values now emerging in Canada, Finland, Germany and Norway; and Yoshino *et al.* [35] reviewed minimum ventilation rates in fifteen developed countries. Finnish Building regulations have specific requirements for IAQ, setting maximum absolute CO_2_ levels at 1200 ppm, along with maximum permissible levels for other pollutants such as sulphur dioxide, nitrogen dioxide, particulates, lead, carbon monoxide and benzene [36]. Germany and Switzerland set a maximum absolute CO_2_ level of 1500 ppm, Germany DIN 1946 Part 6 requires 30 m^3^/h for 2 person flats.

Some studies have been carried out to investigate IAQ in modern energy efficient buildings with natural ventilation strategies, with and without intermittent extract fans. A study by Offerman in California investigated window and ventilation use and reported very low air change rates (average 0.26 ach) with 67% of homes non-compliant with local building codes. In the UK, three Government funded studies carried out by BRE in 2001 [37], 2002 [38] and 2005 [39] found high levels of VOCs with the third study reporting average winter ventilation rates of 0.44 ach. These studies also highlighted that flats were less well ventilated than other house types and that occupant interaction with trickle ventilators was erratic, with only four out of 37 cases where they were maintained in the open position, with 13/37 being permanently closed. Homes with the lowest ventilation rates in winter had the trickle ventilators fully closed. A more recent study [40] monitored 22 dwellings in England found installed trickle ventilator opening areas were less than specified. The study highlighted particular problems in flats-where there is usually less opportunity for cross-flow or displacement ventilation-with acceptable IAQ resulting only where good specification and installation practices were evident and occupants ensured vents were habitually in the open position.

The above summarises only a subset of available literature but serves to illustrate that absolute CO_2_ levels and ventilation rates are beginning to emerge in building regulations, and highlights a range of previous studies into how occupants interact with ventilation components, and how indoor air quaility was measured, these points served to inform the current study.

## 2. Methods

The research was targeted at winter time conditions when low exterior temperatures, high rainfall, high occupancy, and reduced ventilation rates, would provide a worst case situation. Both the occupant interaction survey of 200 dwellings to establish understanding and use of ventilation equipment, and the 40 dwelling monitoring survey were conducted between January and March 2014.

The research specifically targeted dwellings which had been specified and built to have the most common “natural ventilation” option as allowed by the regulations, as stated earlier this “natural ventilation” option requires trickle ventilators in window frames as the main mechanism for background ventilation in all rooms but also includes intermittent extract fans in wet rooms such as kitchens and bathrooms. The research excluded properties with continuous whole house mechanical ventilation systems such as Mechanical Ventilation with Heat Recovery (MVHR) or decentralised mechanical extract ventilation (dMEV).

### 2.1. Occupant Interaction Survey

The first phase of the physical research was to investigate occupant interaction with natural ventilation equipment and this was achieved through a survey conducted by short scripted interview of occupants of 200 newly built homes in Scotland, all constructed to post-2009 regulations.

The survey was carried out at the targeted dwellings and was designed to determine the occupants understanding and use of ventilation equipment. A social survey company with experience of dealing with tenants and occupiers of housing was engaged to carry out the survey. The survey covered the understanding and use of trickle ventilators, window openings, curtains and blinds, and doors in both living areas and bedrooms, there were also further questions on understanding and use of intermittent extract fans. The survey was supplemented with pictures, e.g., showing trickle ventilators *etc.* the interview covered each element of ventilation equipment in turn. For trickle ventilators the occupants were first asked if they were aware they had trickle ventilators, then shown photographs to confirm understanding, they were then asked to confirm the normal position of the trickle vents in their living rooms and bedrooms for various periods of the day and when these rooms were occupied and unoccupied, how often they adjusted the trickle vents, what motivated or prevented their adjustments and settings, and whether and to what extent the ventilators were occluded by blinds, curtains or other window dressings. Similar questions supported by photographs were asked of window openings and door openings to ascertain the behaviours with regard to these ventilation elements, the window and door opening extent was also assessed. Behaviour regarding the use of window coverings such as curtains and blinds was gathered.

In addition to specific information regarding use of ventilation equipment, supplementary questions were asked such as: have you received advice on using trickle ventilation? Have you received advice on ventilating your dwelling? How do you feel about the air quality in your dwelling?

The occupant interaction survey process also asked the occupants if they would be willing to have some measurements made in their dwelling as part of the research project.

### 2.2. Monitoring of Sample Buildings

From those identified as willing to participate in monitoring, a sub-set of 40 houses were identified, representative of the overall population, for a 48hr monitoring study to capture a snapshot of indoor environmental conditions. Measurements were made of temperature, relative humidity (Gemini Tinytag TGU 4500: (−25 to +85 °C/0 to 95% RH)) and CO_2_ (Gemini Tinytag TGE-0011: 0–5000 ppm (−25 to +85 °C/0 to 95% RH) all at 5-min intervals, in both the main living room and the main bedroom. Equipment deployment for these measurements involved a short visit, during this visit the initial interaction survey results were confirmed or updated and additional data and pictorial information was gathered including: house type; sensor locations; room dimensions; room occupancy; orientation and position of windows, trickle vents, doors; occlusion to trickle vents; door undercut size; and possibility of cross ventilation. The size and position of the trickle ventilators, and open/closed extent was recorded and occupants were asked to maintain their trickle ventilator in this position to avoid the Hawthorne effect [41]. The 48-h period was considered sufficient to give a snapshot of ventilation performance in a period where behavior such as occupancy and ventilation equipment settings could be more certain than during a longer measurement period.

## 3. Occupant Interaction with Ventilation Equipment

How occupants responded to questions on use of the trickle ventilators in the bedrooms and living rooms gave a consistent picture, in the majority of cases trickle ventilators were stated to be closed and never opened, in living rooms (63%), and bedrooms (63%). A minority have trickle ventilators always open and never closed in living rooms (24%) and bedrooms (28%). The smallest group were found to adjust them weekly, in living rooms (13%) and bedrooms (9%). No respondents reported that they altered trickle ventilator settings on a daily basis. The vast majority of respondents reported that they never adjusted their trickle ventilators.

**Figure 1:**
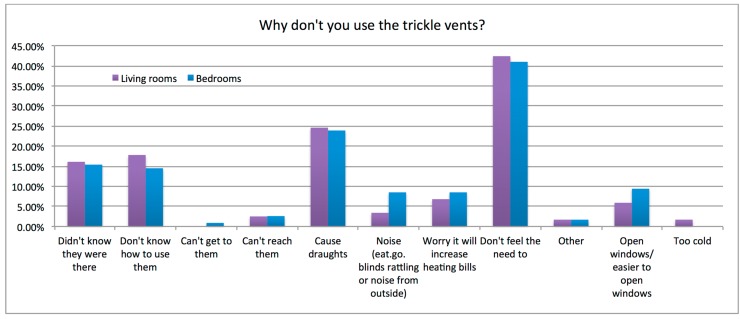
Barriers to trickle ventilators use, living rooms and bedrooms.

Reasons for not interacting with trickle ventilators are summarized in Figure 1. The largest response was from occupants who reported that they “don’t feel the need to open vents” (41%). This is backed up by responses to questions about perceived air quality where 92% of respondents described air quality in the bedroom as “good” or “very good”. Other selected reasons for not interacting with trickle ventilators were lack of knowledge/awareness (16%, 16%, total 32%), that they cause draughts (24%), it is easier to open a window (7.5%), and increased heating bills (7.5%).

Responses to questions about window opening were more diverse than for trickle ventilators. Some responded that they never opened windows, in living rooms (22%) and bedrooms (16%). Most reported opening windows a few times a week (33%, 34% for living rooms and bedrooms respectively). Others reported opening once or more per day (30%, 32% respectively). This occasional window opening is somewhat consistent with the assumption in regulations that windows are for “purge” ventilation in that the majority of occupants reporting window opening events at least a few times per week (63%, 66%). An additional 12% reported living room windows were always open and 19% that bedroom windows were always open.

**Figure 2:**
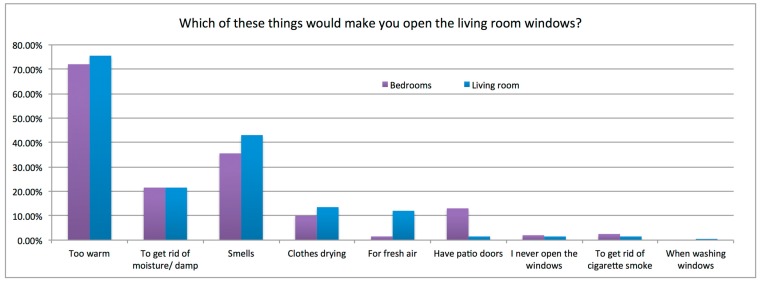
Drivers for window opening, living rooms and bedrooms.

Occupant responses on the drivers for a window opening event are summarized in Figure 2. “Too warm” was identified by many occupants as a situation that would motivate a window opening event (75% living rooms, 72% bedrooms), other motivations included moisture control, smells, clothes drying, and a desire for fresh air. Barriers that would act to inhibit window opening were reported as “heat loss” (59%), noise (17%), security (11%) and pollution (5%).

Responses to questions on door opening and curtain and blind usage provided more insight for bedrooms due to their simpler geometry and use patterns than living rooms (open plan, multiple internal and external doors, complex user patterns). It was reported that bedroom doors were fully closed in 40% of cases, open a little in 44% of cases and fully open in 16% of cases. Bedroom blinds and/or curtains were mostly closed when sleeping (88%).

82% of the respondents stated they had not received advice on how to best ventilate their house. Of the 18% who stated they received advice 40% of them stated that the advice had been to leave trickle ventilators open.

## 4. IAQ Measurements for Sample Dwellings

The monitoring was undertaken at seven geographical locations across Scotland. Dwelling types were a mixture of flats (62%), two storey houses (34%) and single storey houses (4%). All properties had at least two windowed elevations allowing the possibility of cross ventilation from front to back (62%) or front to side (38%) assuming internal doors and windows or vents are open. Construction types were a mixture of timber frame, masonry and steel frame, all incorporating intermittent mechanical ventilation to bathrooms and kitchens, and with trickle ventilators positioned in window frames of every room. The trickle ventilators were generally located 2 m above floor level in the upper element of the window frame. The monitored dwellings were selected based on the willingness of participants and also to cover the range of conditions identified in the earlier occupant survey.

These specific post-2009 dwellings had not had their air-tightness tested however similar dwellings within the same developments had been tested and data submitted as part of the regulatory process with an average measured air-tightness of 4.0 m^3^/hr/m^2^ @ 50 Pa. Weather data including average wind speeds was recorded e.g., average overnight wind speed during the measurements was 3.3 m/s with a standard deviation of 1.02 m/s. Average temperature was 7.6 °C with a standard deviation of 2.4 °C.

**Figure 3:**
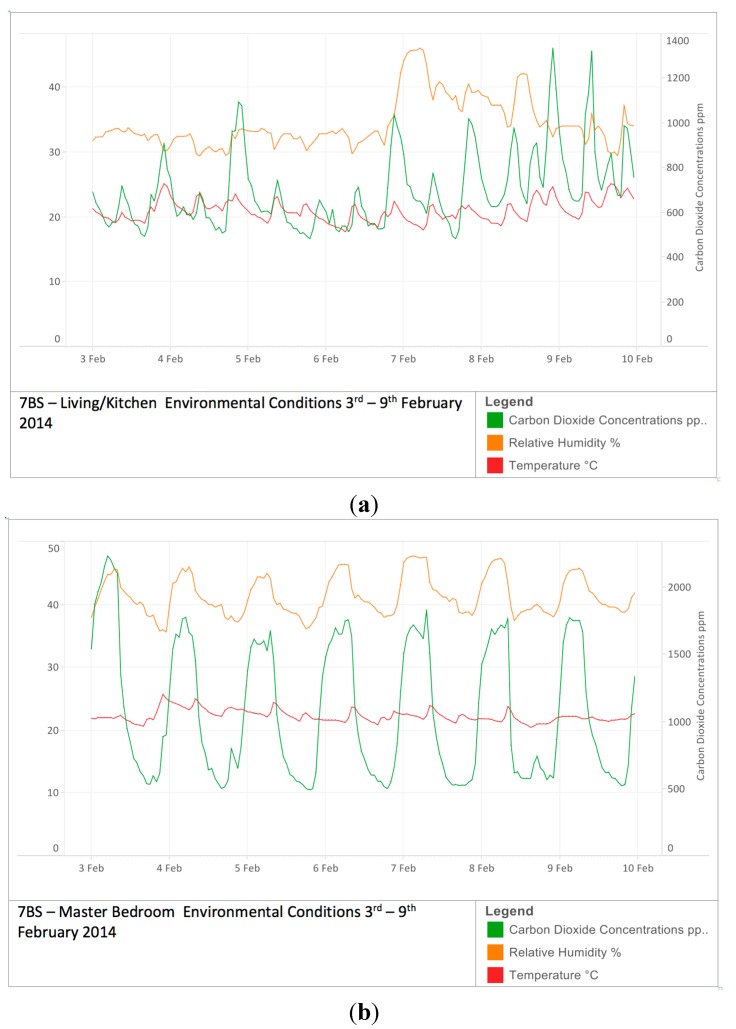
(**a**) Temperature, Relative Humidity and CO_2_ levels living room (**b**) Temperature, Relative Humidity and CO_2_ levels bedroom.

While temperature, humidity and CO_2_ concentrations were measured for both living rooms and bedrooms (Figure 3), focus here is on CO_2_ levels in bedrooms. CO_2_ levels are a proxy for ventilation rates when occupancy is known, and bedrooms due to their simpler circumstances (geometry, user patterns, occupancy *etc.*) support clarity of insight, allowing ventilation strategies to be compared.

For the measured dwellings: 77% of dwellings kept bedroom windows closed at night, 55% kept bedroom doors closed at night and 53% had both windows and doors closed. A total of 64% had bedroom trickle ventilators closed at night. All the dwellings had some form of occlusion to the trickle ventilators at night, in the form of curtains and/or blinds. All bedrooms monitored were classified as double bedrooms with an average floor area of 12 m^2^ and an average volume of 28.6 m^3^. The number of occupants ranged from a single person up to a maximum of six people where occupancy greater than two was due to children. Three occupancy categories considered were one person (40% of cases); two persons (47%); three + persons (10%).

### 4.1. Measured Bedroom CO_2_ Levels

Much detailed information was gathered. Two metrics in particular are used here to illustrate the main findings: (i) the average bedroom CO_2_ levels during the period 11 pm to 7 am (11–7); and (ii) the percentage of time during the 11 pm to 7 am period that CO_2_ concentration exceeds 1000 ppm.

**Figure 4:**
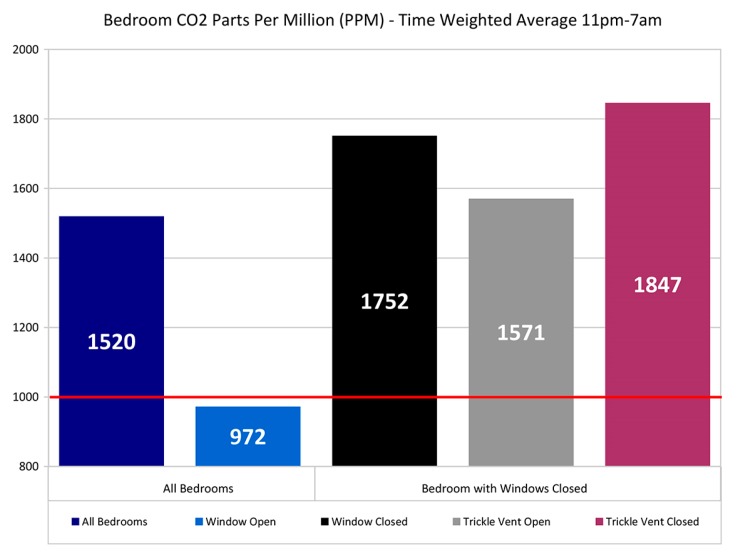
Bedroom CO_2_ ppm time weighted average 11 pm–7 am.

The overall average of the average 11–7 bedroom CO_2_ levels for all dwellings with complete data (*n* = 39) was 1520 ppm as shown in Figure 4. With open windows (*n* = 11) the overall average CO_2_ is 972 ppm, with windows closed (*n* = 28) it is 1752 ppm. With windows closed and open trickle ventilators CO_2_ levels were to 1571 ppm compared with vents closed at 1847 ppm but the overall impact of trickle ventilator position appears relatively small compared to window position.

**Figure 5:**
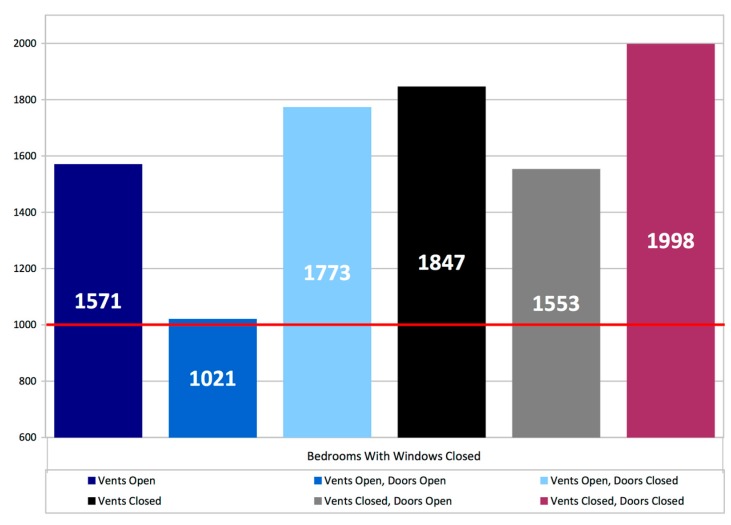
Bedrooms with windows closed-average CO_2_ ppm, time weighted average between 11 pm and 7 am.

Further breakdown of the results for different door and trickle ventilator settings when bedroom windows are closed is shown in Figure 5. In closed window bedrooms with both door and trickle ventilators closed the average 11–7 measured absolute CO_2_ values were 1998 ppm. In closed window bedrooms with either doors OR trickle ventilators open values were 1553 ppm and 1571 ppm respectively. In closed window bedrooms with both doors AND trickle ventilators open average measured values were 1021 ppm. Some caution needs to be expressed here due to low sample size (*n* = 3) however it appears that the combined effect of trickle ventilation PLUS door opening is much greater than either measure on its own, potentially pointing to enhanced ventilation rates with more than one ventilation opening per room, an avenue for more study. The average door undercut was recorded was 10 mm, with a third of bedrooms having no door undercut.

**Figure 6:**
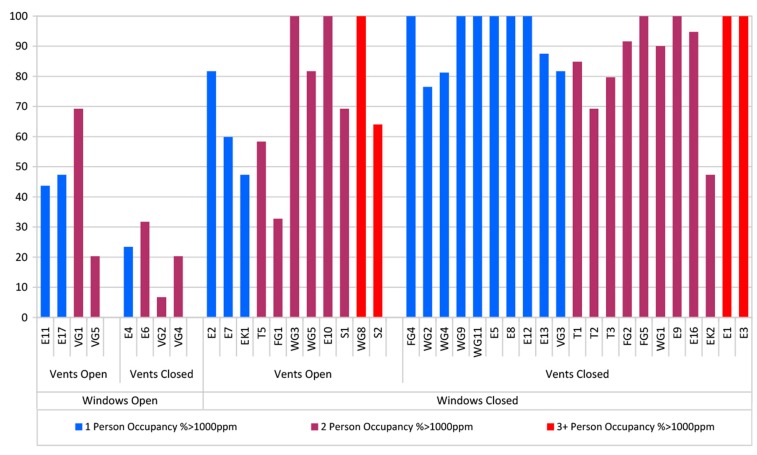
Percentage of time bedroom CO_2_ levels exceed 1000 ppm between 11 pm and 7 am across all of the 40 monitored dwellings.

These comparisons of averages serve to illustrate the findings for the different ventilation equipment settings but the 11 pm –7 am averages across all properties do not capture the property to property variations or the variation in measured CO_2_ levels within the 11 pm–7 am periods. Figure 6 is an attempt to illustrate these details and shows for each monitored dwelling the percentage of time between 11 pm and 7 am that individual bedrooms measured absolute CO_2_ is in excess of 1000 ppm. The dwellings are grouped by category: with and without windows open, with and without vents open, and by occupancy (Dwellings, E11, E17, E4, E2, EK1, FG4, WG2,4,9,11, E5,8,12,13, and VG3 have single occupancy, WG8, S2, E1,3 have three + occupancy, all others have two person occupancy).

All properties, regardless of window position, have CO_2_ in excess of 1000 ppm at some point during the two night monitoring period. Where windows are left open overnight the effects on CO_2_ levels are obvious with these eight dwellings, showing significantly lower periods above 1000 ppm. In the sub-set where windows are permanently closed, the trickle ventilators open condition shows a small improvement in performance, but CO_2_ levels are over 1000 ppm for a significant portion of the overnight period. The dwellings with windows closed, vents and doors open are T5, FG1 and S2 which appear to perform better than similarly occupied dwellings where the doors are closed. The effects of occupancy are less clear.

While the sample size and the short monitoring period provide a snap-shot of the situation in Scottish dwellings to post-2009 building regulations which utilize common “natural ventilation” option with trickle ventilators in window frames and intermittent extract fans in wet rooms, the results provide a valuable insight where previously there was none. The summarized analysis here is primarily concerned with overall trends and not individual peaks, however it was noted that peak absolute CO_2_ concentrations of 5000 ppm were being reached in some instances.

### 4.2. Ventilation rates Based on CO_2_ Measurements

A set of simplified calculations were used to explore the relationship between CO_2_ levels, overall ventilation rate, and other factors such as occupancy. The term “overall ventilation” is used here to represent the sum of both intended ventilation (fresh air input through designed openings such as windows and doors) and unintended or “fortuitous” ventilation (infiltration through unintended construction leakages such as incomplete window seals *etc.*). These calculations required assumptions to be made where data was not measured and therefore the outputs should be viewed as illustrative rather than absolute. The calculations use the standard equation [42] shown in Equation (1) relating the overall ventilation rate, to the equilibrium indoor CO_2_ concentration for a given outdoor concentration:

(1) $$Qv=\frac{1}{Ev}\cdot \frac{G}{\left(Ci-Co\right)}$$

Here *Qv* is the overall ventilation rate, *Ev* is the ventilation effectiveness (assumed to be 1 representing full mixing), *Ci* is the indoor equilibrium CO_2_ concentration, *Co* is the outdoor and background CO_2_ concentration (374 ppm used here) and *G* is the indoor CO_2_ generation rate. This indoor generation rate *G* is a function of the number of room occupants *N,* (this number of bedroom occupants was taken from survey responses) and their metabolic CO_2_ production rates as given in Equation (2) [43]:

(2) $$\text{G}=N\cdot \left(4\;\times \;10^{-5}\right)\cdot M\cdot A$$

*M* in the equation represents the metabolic rate of the occupants in the bedrooms. Metabolic rate varies with activity *i.e.*, 40 W/m^2^ of body surface area when sleeping, 58 W/m^2^ when reading, 116 W/m^2^ when walking, *etc.* [44]. To capture the mix of non-sleep activity and the various phases of sleep activity, a metabolic rate of 60W/m^2^ body surface area was used. *A* is the average body surface area of the person (assumed to be a standard 1.8 m^2^ here).

These simplified calculations were used to determine the overall ventilation rate and overall ventilation rates per person from the estimated CO_2_ generation rate and measured CO_2_ equilibrium concentrations. Per person overall ventilation rates were then calculated using the occupancy. The average calculated per person overall ventilation rate was 3.1 L/s/p and the range was from 0.9 L/s/p (one person, doors, vents and windows closed) to 6 L/s/p (two persons, windows closed, door and vents open) however no dwellings appeared to achieve the commonly stated desired rate of 8 L/s/p.

Overall bedroom air change rates were then calculated from the overall ventilation rate together with the bedroom volumes recorded during the surveys. The average calculated air change rates were 0.7 ach, with 42% of properties having calculated air change rates of less than 0.5 ach, the commonly stated minimum rate required to control moisture.

**Figure 7:**
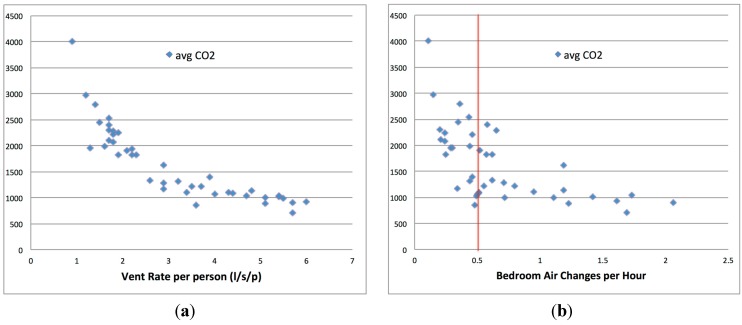
(**a**) Average bedroom CO_2_ level v. ventilation rate per person (**b**) Average bedroom CO_2_ level v bedroom air change rate.

The average measured night time CO_2_ levels in bedrooms plotted against calculated per person overall ventilation rates and bedroom overall air change rates are shown in Figure 7a,b. The strong relationship between CO_2_ levels and these two parameters is clearly evident. The simplified nature of these calculations, and assumptions such as the metabolic rates of individuals, occupancy, outdoor CO_2_ levels *etc.*, will add some uncertainties, but in the main the governing relationships are clear and well established.

## 5. Discussion

### 5.1. Adaptive Response

Although the majority of occupants are aware of the purpose of trickle ventilators, there is relatively little occupant interaction with most remaining permanently closed. Windows are the preferred method for perceived reactive ventilation provision and, in the main this appears to be as a means to control temperature and odours or moisture rather than air quality. The majority of participants did not feel the need to open trickle ventilators as they did not perceive IAQ as being problematic. Changes in air quality are relatively gradual and it is therefore difficult for occupants to perceive a progressive reduction in quality and build-up of CO_2_, due to human adaptation responses that accommodate and suppress reactions to prolonged stimuli. It requires most individuals to leave and re-enter a room to experience the sudden change in IAQ conditions that can trigger a reactive response such as window opening.

### 5.2. Bedroom Conditions

Such human responses become even more acute in the bedroom where occupants may go to bed in IAQ conditions that are reasonable, but progressively deteriorate overnight when the occupants are asleep, especially where bedroom doors are closed and trickle ventilators are either shut, due to external noise, or occluded by blinds and/or curtains. In general, good ventilation rates are not being achieved in the majority of bedrooms. Whilst window opening is a clear mitigating factor–and there are examples where open trickle ventilators in conjunction with open bedroom doors improved ventilation and slowed the rate of CO_2_ increase–all bedroom occupants in the study were subjected to periods where CO_2_ levels were significantly above 1000 ppm, with the many dwellings averaging close to twice the threshold. The closing of vents and doors will create predictable barriers to ventilation, but given that both these element are closeable, this must be considered as predictable behavior, especially when driven by self-closer mechanisms, as required by fire regulations in some flatted developments.

### 5.3. Ease of Use

It was observed that trickle ventilators are frequently out of immediate reach–particularly for the elderly-due to height, furniture and positioning of blinds and curtains. The placement of trickle ventilator controls differs from window handles, which are required to be in a more accessible position. There are few homes where windows do not have some form of device that can provide the occupants with both privacy and blackout conditions to assist with sleep. Although the effects of occlusion was not measured in this study it is possible that these will inhibit air movement. The fact that air has to travel in both directions (where cross ventilation is not an option) further reduces the efficacy of a trickle ventilator.

### 5.4. Cross Ventilation

The reliance on door opening to achieve adequate ventilation is also a cause for concern. Observations from the study suggest the number and age of occupants in the household affect door-closing habits. For example, where a young child is present doors are almost always open. The elderly also tend to keep doors open especially when challenged by mobility or health issues. Conversely, it is more likely that a teenager will seek privacy. Other factors leading to door closure include noise, concerns about fire (or in some cases self-closers) and security.

In the majority of cases door undercuts that might be intended to assist cross ventilation were ineffective, either because they had not been provided or because the installation of floor coverings frequently obstructed them, with some residents reporting having to physically shorten doors after fitting carpets; and that air movement under doors may be perceived as draughts. This indicates a lack of understanding about the potential for cross ventilation within the home.

This is further complicated by residents’ inability to determine good air quality. The questionnaire indicated that over 90% of respondents, describe the air quality in their master bedroom as very good or fairly good. This contradicts the results from the physical surveys where 83% of properties present with time weighted average CO_2_ concentrations (11 pm–7 am) in bedrooms greater than 1000 ppm.

### 5.5. Energy Use versus IAQ

The general picture emerging from this research illustrates the increasing tensions that exist between energy efficiency and IAQ, with concern about heat loss being reported as the prime reason for keeping vents and windows permanently closed. The evidence suggests that adaptive behavior due to temperature, moisture, odour or seasonal habits, leads to window opening rather than trickle ventilator use. The difficulty here is that while purge ventilation can deal with the immediate condition, occupied internal environments will begin to degrade as soon as windows are closed. The monitoring has shown worryingly high CO_2_ levels in bedrooms and the resulting calculated air change rates indicate likely problems with internal relative humidity producing condensation, mould growth and house dust mite colonisation and proliferation [45].

## 6. Conclusions

These findings support a view that adherence to the guidance for provision for natural ventilation as laid down in the Building Standards is not resulting in desirable levels of ventilation. In the main, trickle ventilators are not being used by the occupants as envisaged, but even where they are permanently open, they do not ensure sufficient background ventilation to ensure healthy IAQ.

The use of trickle ventilators does improve ventilation rates, as does internal door opening, but not to a reliable standard that would maintain CO_2_ levels below the recognised threshold representing reasonable IAQ. The current regulatory guidance is thus very sensitive to fortuitous effects and does not account for known variables including the occupancy level, built form and availability of cross ventilation. Ventilation rates are further reliant upon (and sensitive to) uncontrolled factors including external weather, internal door opening, and occlusion of vents by curtains and blinds. The use of window opening for purge ventilation is evident, but this does not address a need to maintain minimum background ventilation levels. Whilst adequate ventilation may be achieved by permanent overnight window opening, and it has been estimated that the energy cost of this may be as little as 2% [46], this is not a reliable or robust strategy across all house types due to external factors. A requirement to keep bedroom doors open may not be acceptable, and undercuts to doors are also not effective due to occlusion by floor coverings.

The study suggests that poor ventilation may be endemic in new build dwellings across Scotland. Given the evidence on ventilation and health identified in the literature review, there is a concern that this may have a negative impact on public health, however further research is needed to examine relationships between ventilation and health in housing.

## 7. Recommendations

(1) Develop and adopt a standard testing protocol that would enable an evaluation of an as-built performance standard to demonstrate that acceptable ventilation can be achieved within normal use. This will allow architects and house builders the freedom to apply a range of ventilation strategies that are likely to include, in additive or synergistic combination, cross ventilation, displacement and stack ventilation, wet zone extraction and mechanical positive pressure or balanced regimes, with or without heat recovery. A simple solution to the problem may be to simply increase internal volumes.

(2) Provide occupants with better information about ventilation in the home. This may include improved guidance at hand-over stages, but could also include use of CO_2_ monitors to help occupants identify when poor IAQ is being experienced to stimulate adaptive behavior beyond thermal comfort.

(3) Undertake a major longitudinal and epidemiological study to measure the potential health impacts of poor IAQ and relate this to measured levels of CO_2_ in buildings.

## References

[1] Palmer J., Cooper I. Great Britain’s Housing Energy Fact File 2011. http://www.cewales.org.uk/cew/wp-content/uploads/Great-Britains-Housing-Energy-Fact-File-2011.pdf.

[2] The Building Regulations 2010. http://www.planningportal.gov.uk/uploads/br/BR_PDF_AD_L1A_2013.pdf.

[3] The Scottish Building Regulations. https://books.glgoo.com/books?hl=zh-CN&lr=&id=xM1WCMrs0hkC&oi=fnd&pg=PR5&dq=Scottish+Building+Regulations&ots=NnZYlvfHlL&sig=pr3335RfuxteCw2oWhovJG2aiu0&redir_esc=y#v=onepage&q=Scottish%20Building%20Regulations&f=false.

[4] Stephen R.K. (1998). Airtightness in UK Dwellings: BRE’s Test Results and Their Significance.

[5] Stephen R.K. Airtightness in UK Dwellings. http://www.opengrey.eu/item/display/10068/654323.

[6] Grigg P. Assessment of Energy Efficiency Impact of Building Regulation Compliance. http://www.ukace.org/wp-content/uploads/2007/10/2004-11-10-Assessing-of-Energy-Efficiency-Impact-on-Building-Regulations-Compliance.pdf.

[7] Wingfield J., Bell M., Bell J., Lowe R. Evaluating the Impact of an Enhanced Energy Performance Standard on Load-Bearing Masonry Construction. http:/www.communities.gov.uk/documents/corporate/pdf/2219033.pdf.

[8] Airtightness of UK Housing. http://www.leedsmet.ac.uk/teaching/vsite/low_carbon_housing/airtightness/housing/housing.pdf.

[9] Crump D., Dengel A., Swainson M. (2009). Indoor Air Quality in Highly Energy Efficient Homes—A Review.

[10] Davies M., Oreszczyn T. (2012). The unintended consequences of decarbonising the built environment: A UK case study. Energ. Buildings.

[11] Technical Handbook, Section 6: Energy. http://www.gov.scot/Topics/Built-Environment/Building/Building-standards/techbooks/techhandbooks/th2015dom6.

[12] Phillips T., Rogers P., Smith N. Ageing and Airtightness-How Dwelling Air Permeability Changes Over Time. http://products.ihs.com/cis/Doc.aspx?AuthCode=&DocNum=296303.

[13] The Effect That Increasing Air-Tightness May Have on Air Quality Within Buildings. http://www.scotland.gov.uk/Resource/0040/00402329.pdf2012.

[14] Howieson S.G., Sharpe T., Farren P. (2014). Building tight–ventilating right? How are new air tightness standards affecting indoor air quality in dwellings?. Build. Serv. Eng. Res. T..

[15] Hasselar E. (2006). Health Performance of Housing Indicators and Tools.

[16] Hasselar E., van Ginkel J.T. How Healthy Is the Bedroom?. http://www.researchgate.net/publication/27347574_How_healthy_is_the_bedroom.

[17] Schell M., Int-Hout D. (2001). Demand Control Ventilation Using CO_2_. ASHRAE J..

[18] Locher W.G. (2007). Max von Pettenkofer (1818–1901) as a Pioneer of Modern Hygiene and Preventive Medicine. Environ. Health Prev. Med..

[19] Porteous C.D.A. Sensing a Historic Low-CO_2_ Future. http://cdn.intechopen.com/pdfs/16326/InTech-Sensing_a_historic_low_co2_future.pdf.

[20] Appleby A. (1990). Indoor Air Quality and Ventilation Requirements. Buildings and Health, the Rosehaugh Guide to the Design, Construction, Use and Management of Buildings.

[21] Seppänen O.A., Fisk W.J., Mendell M.J. (1999). Association of ventilation rates and CO_2_-concentrations with health and other responses in commercial and institutional buildings. Indoor Air.

[22] Erdmann C.A., Steiner K.C., Apte M.G. Indoor carbon dioxide concentrations and sick building syndrome symptoms in the base study revisited: analyses of the 100 building dataset. http://escholarship.org/uc/item/1mf005ws.

[23] Batterman S., Peng C. (1995). TVOC and CO_2_ concentrations as indicators in indoor air quality studies. Am. Ind. Hyg. Assoc. J..

[24] Indoor Air Quality and Ventilation (CIBSE Knowledge Series). http://www.abebooks.com/9781906846190/Indoor-Air-Quality-Ventilation-CIBSE-1906846197/plpISBN9781906846190.

[25] Standard 62.1 Ventilation for Acceptable Indoor Air Quality 2013. https://www.ashrae.org/resources--publications/bookstore/standards-62-1--62-2.

[26] CIBSE (2006). Guide A: Environmental design.

[27] Indoor Environmental Input Parameters for Design and Assessment of Energy Performance of Buildings Addressing Indoor Air Quality, Thermal Environment, Lighting And Acoustics. http://shop.bsigroup.com/ProductDetail/?pid=000000000030133865.

[28] Ventilation in Homes: A Breath of Fresh Air?. http://www.zerocarbonhub.org/sites/default/files/Occupant%20Ventilation.pdf.

[29] ASHRAE 62.2 FAQs. http://waptac.org/Additional-Pages/FAQ-ASHRAE-62002E2.aspx.

[30] EU EnVIE WP3 Technical Report: Characterisation of Spaces and Sources. http://cordis.europa.eu/documents/documentlibrary/126459681EN6.pdf.

[31] Offermann F.J. Ventilation and Indoor Air Quality in New Homes. http://www.arb.ca.gov/research/apr/past/04-310.pdf.

[32] Ramalho O., Wyart G., Mandin C., Blondeau P., Cabanes P.A., Leclerc N., Redaelli M. (2015). Association of carbon dioxyde with indoor air pollutants and exceedance of health guideline values. Indoor air.

[33] Kim C.S., Lim Y.W., Yang J.Y., Hong C.S., Shin D.C. (2002). Effects of Indoor CO_2_ Concentrations on Wheezing Attacks in Children. Proc. Indoor Air.

[34] Harrison P.T.C. Guidelines for Indoor Air Quality. http://www.euro.who.int/en/health-topics/environment-and-health/air-quality/policy/who-guidelines-for-indoor-air-quality.

[35] Yoshino H., Murakami S., Akabayashi S.I., Kurabuchi T., Kato S., Tanabe S.I., Adachi M. Survey on Minimum Ventilation Rate of Residential Buildings in Fifteen Countries. http://www.aivc.org/sites/default/files/members_area/medias/pdf/Conf/2004/2004028_Yoshino.pdf.

[36] Ministry of the Environment Housing and Building Department D2, Indoor Climate and Ventilation of Buildings Regulations and Guidelines. www.buildup.eu/sites/default/files/D2eng%20-ventilation%20guidelines%20in%20Finalnd%20%20in%20English_p.pdf.

[37] Coward S.K.D., Llewellyn J.W., Raw G.J., Brown V.M., Crump D.R., Ross D.I. Indoor air quality in homes in England. http://www.opengrey.eu/item/display/10068/671361.

[38] Coward S.K.D., Llewellyn J.W., Raw G.J., Brown V.M., Crump D.R., Ross D.I. Indoor Air Quality in Homes in England–Volatile Organic Compounds. http://www.opengrey.eu/item/display/10068/666475.

[39] Dimitroulopoulou C., Crump D., Coward S.K.D., Brown V., Squire R., Mann H., White M., Pierce B., Ross D. BR 477: Ventilation, Air Tightness and Indoor Air Quality in New Homes. http://www.umad.de/infos/cleanair13/pdf/full_104.pdf.

[40] Ventilation and Indoor Air Quality in Part F 2006 Homes. http://docslide.us/documents/ventilation-and-indoor-air-quality-in-part-f-2006-homes.html.

[41] Schwartz D., Baruch F., Tamar K., Fallaw S. (2013). The Hawthorne effect and energy awareness. P. Natl. Acad. Sci. USA.

[42] European Concerted Action Indoor Air Quality and its Impact on Man, Report No.11, Guidelines for Ventilation of Buildings. http://www.inive.org/medias/ECA/ECA_Report11.pdf.

[43] KS17 Indoor Air Quality and Ventilation (CIBSE Knowledge Series KS17). http://www.cibse.org/knowledge/cibse-ks/ks17-indoor-air-quality-ventilation.

[44] CIBSE Concise Handbook. http://www.cibse.org/knowledge/cibse-guide/concise-handbook.

[45] Howieson S. (2005). Housing and Asthma.

[46] Ucci M., Biddulph P., Oreszczyn T., Crowther D., Wilkinson T., Pretlove S.E., Hart B., Ridley I. (2011). Application of a transient hygrothermal population model for house dust mites in beds: Assessment of control strategies in UK buildings. J. Build. Perform. Simu..

